# Supplementary material for: Revisiting the Tigger Transposon Evolution Revealing Extensive Involvement in the Shaping of Mammal Genomes
Source: Biology (Basel). 2022 Jun 16;11(6):921. doi: 10.3390/biology11060921 (PMC9219625; doi:10.3390/biology11060921)
Supplement: Supplementary file 1 [file biology-11-00921-s001.zip › biology-1735978-supplementary/supplementary/Supplementary figure S2.pdf]

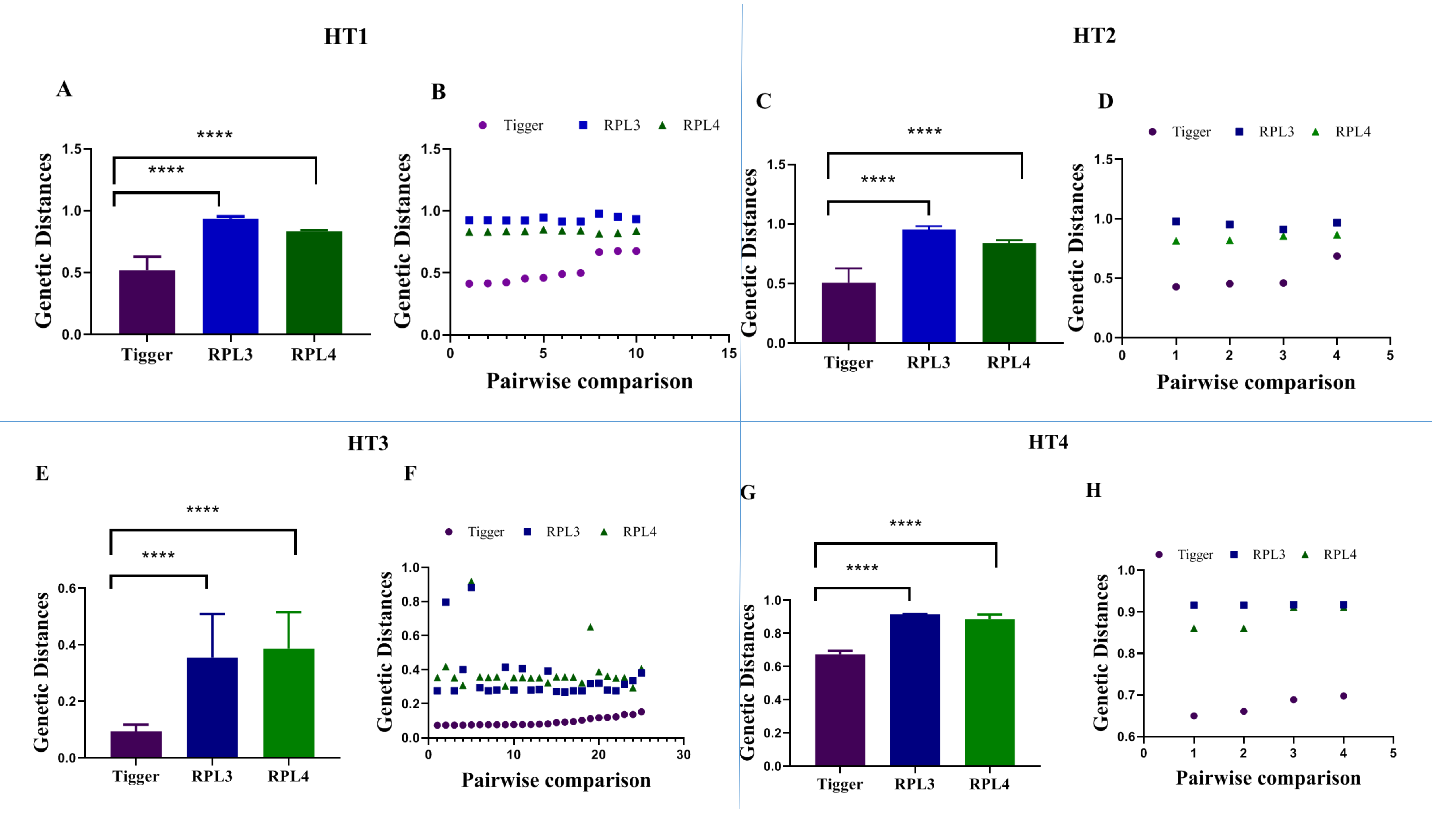

HT1
A
B
C
D
HT2
HT3
E
F
G
H
HT4
Tigger
RPL3
RPL4
Genetic Distances
Pairwise comparison
****

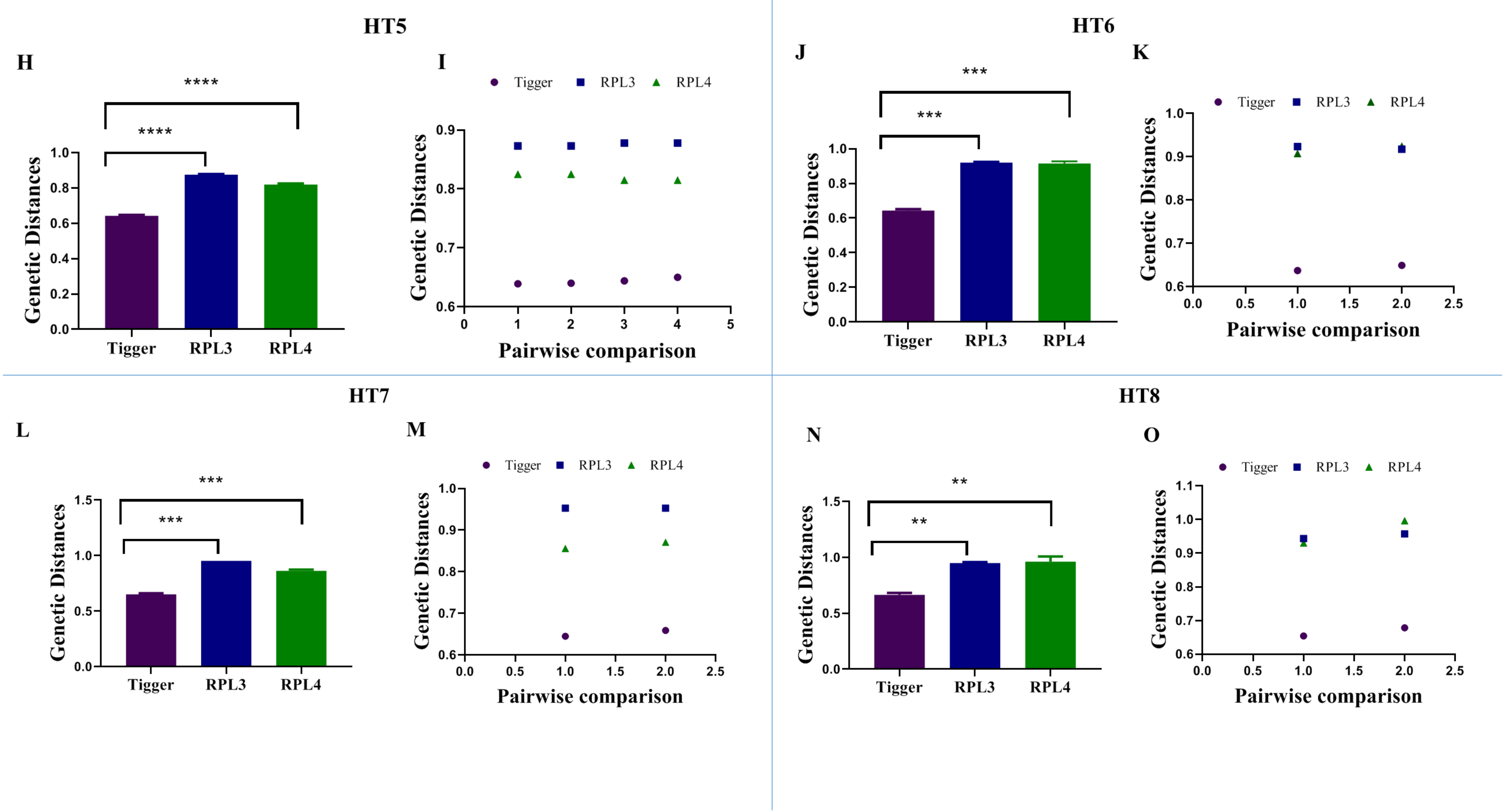

HT5
H
****
****
Genetic Distances
1.0
0.8
0.6
0.4
0.2
0.0
Tigger
RPL3
RPL4
I
Tigger
RPL3
RPL4
Genetic Distances
0.9
0.8
0.7
0.6
0
1
2
3
4
5
Pairwise comparison
HT6
J
***
***
Genetic Distances
1.0
0.8
0.6
0.4
0.2
0.0
Tigger
RPL3
RPL4
K
Tigger
RPL3
RPL4
Genetic Distances
1.0
0.9
0.8
0.7
0.6
0.0
0.5
1.0
1.5
2.0
2.5
Pairwise comparison
HT7
L
***
***
Genetic Distances
1.5
1.0
0.5
0.0
Tigger
RPL3
RPL4
M
Tigger
RPL3
RPL4
Genetic Distances
1.0
0.9
0.8
0.7
0.6
0.0
0.5
1.0
1.5
2.0
2.5
Pairwise comparison
HT8
N
**
**
Genetic Distances
1.5
1.0
0.5
0.0
Tigger
RPL3
RPL4
O
Tigger
RPL3
RPL4
Genetic Distances
1.1
1.0
0.9
0.8
0.7
0.6
0.0
0.5
1.0
1.5
2.0
2.5
Pairwise comparison

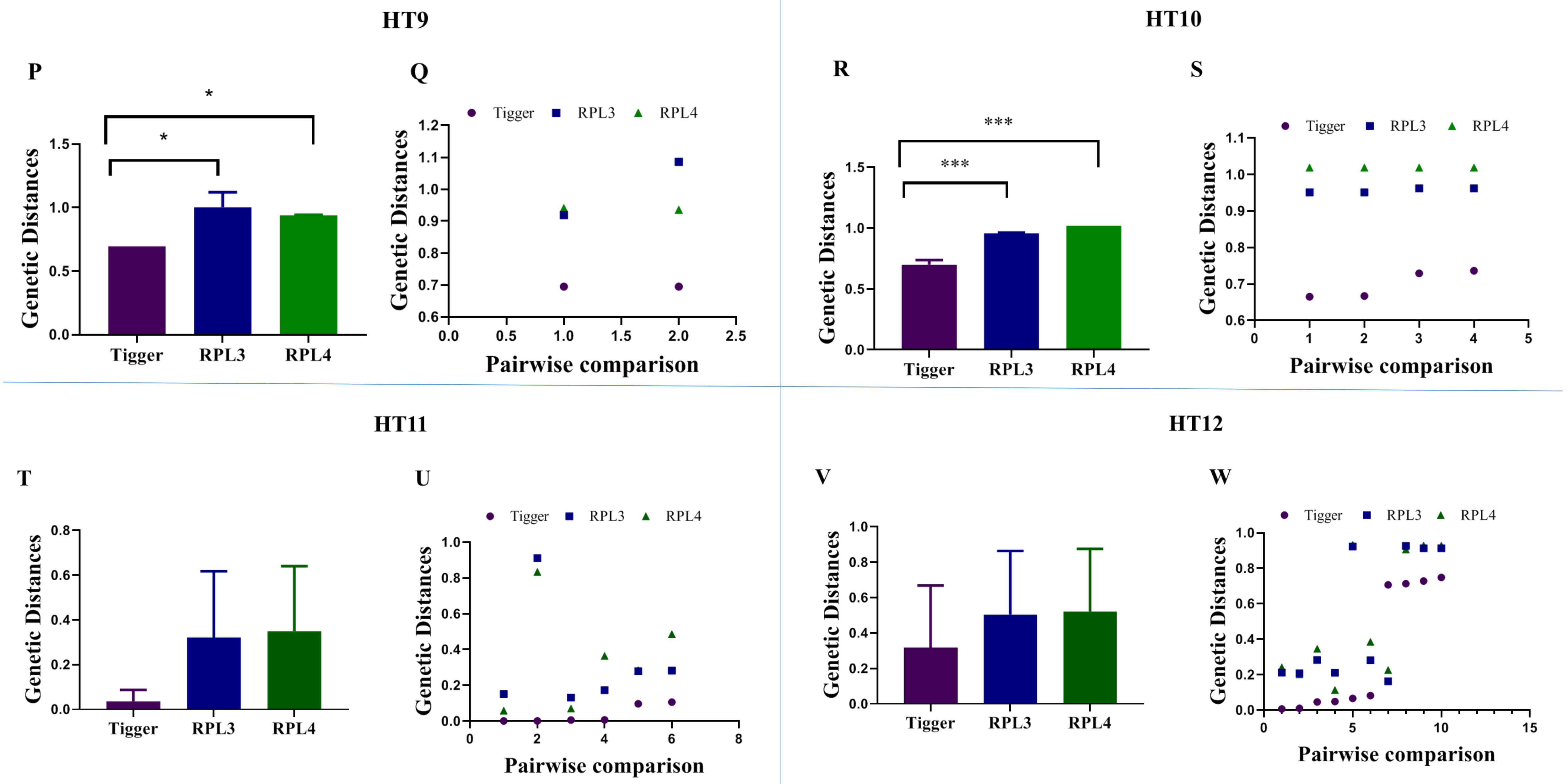
HT9
HT10
HT11
HT12
P
Q
R
S
T
U
V
W
*
***
Tigger
RPL3
RPL4
Genetic Distances
Pairwise comparison

HT13

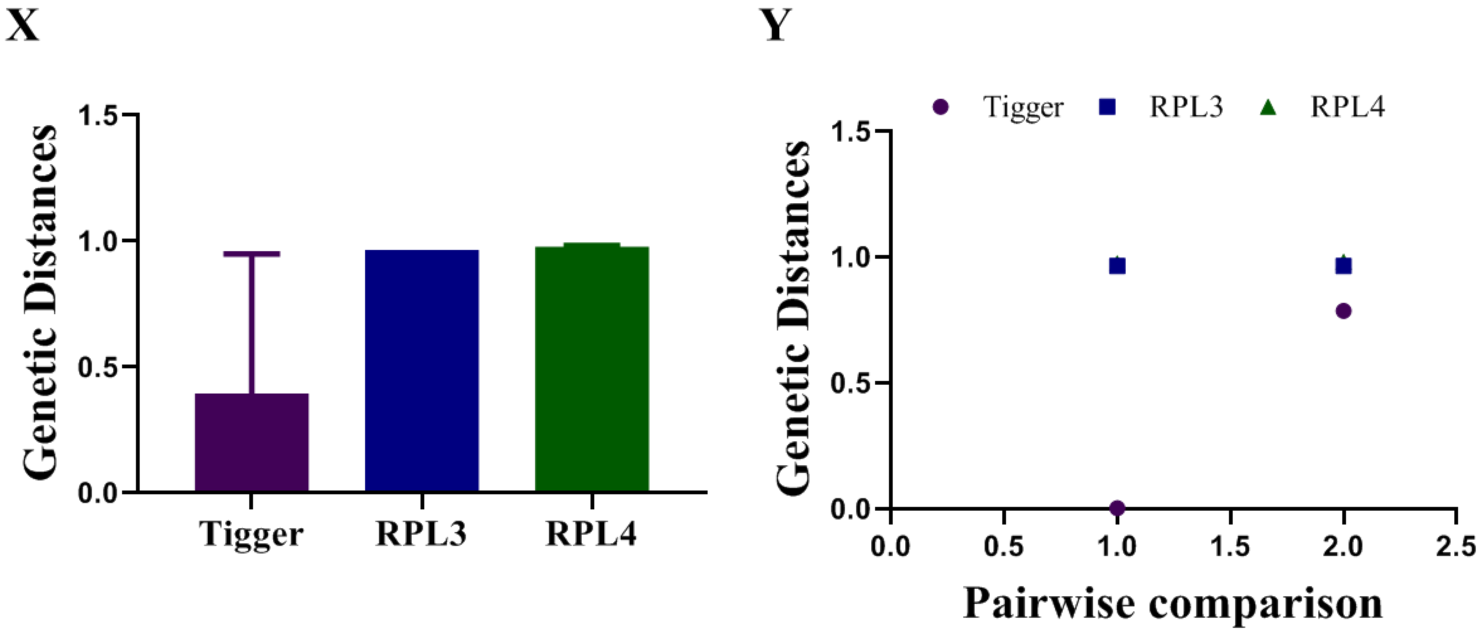

Figure S2: Genetic distances analysis for *Tigger* transposons and host genes. The graph illustrates the pairwise distances of *Tigger* and two host genes between the species involved in HT1, HT2, HT3, HT4, HT5, HT6, HT7, HT8, HT9, HT10, HT11, HT12, and HT13 events respectively. The distances were obtained from different phyla or classes pairwise comparisons between the species in which *Tigger* was identified.
