# Supplementary material for: Revisiting the Tigger Transposon Evolution Revealing Extensive Involvement in the Shaping of Mammal Genomes
Source: Biology (Basel). 2022 Jun 16;11(6):921. doi: 10.3390/biology11060921 (PMC9219625; doi:10.3390/biology11060921)
Supplement: Supplementary file 1 [file biology-11-00921-s001.zip › biology-1735978-supplementary/supplementary/Supplementary figures S3 & S4.pdf]

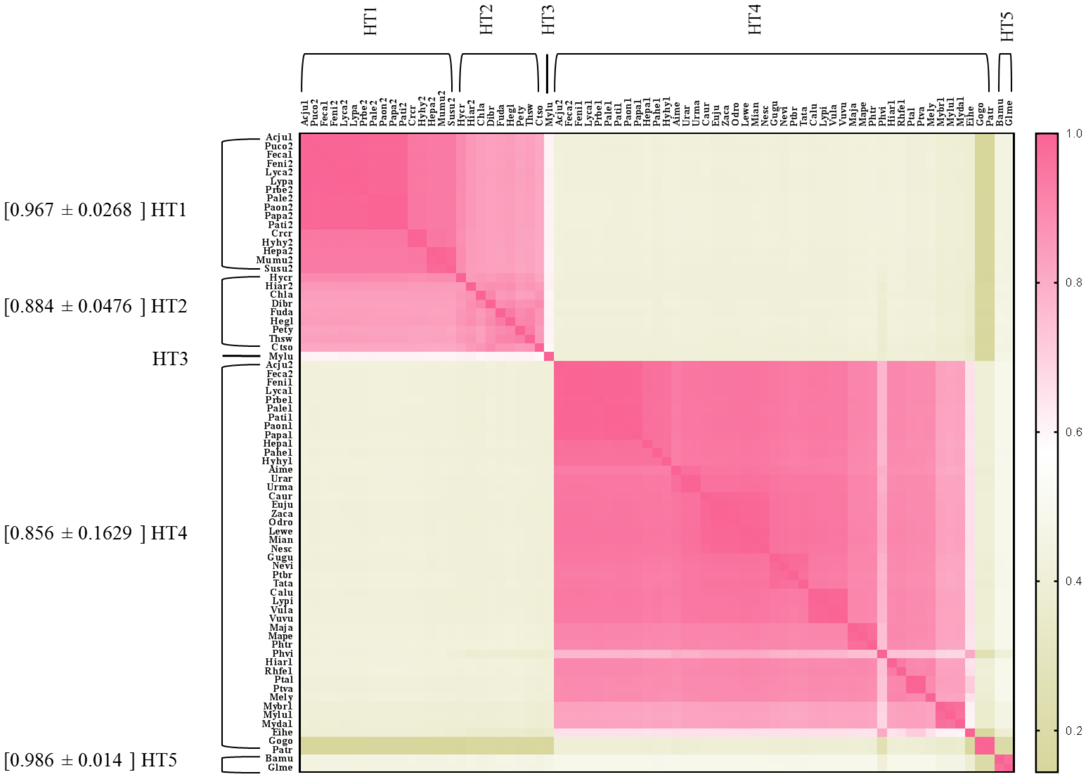

Figure S3: Sequence identities between *Tigger* elements among mammalian species. The sequence identities were measured by pairwise comparisons of *Tigger* CDS sequences (for species abbreviations refer to Supplementary table S1).

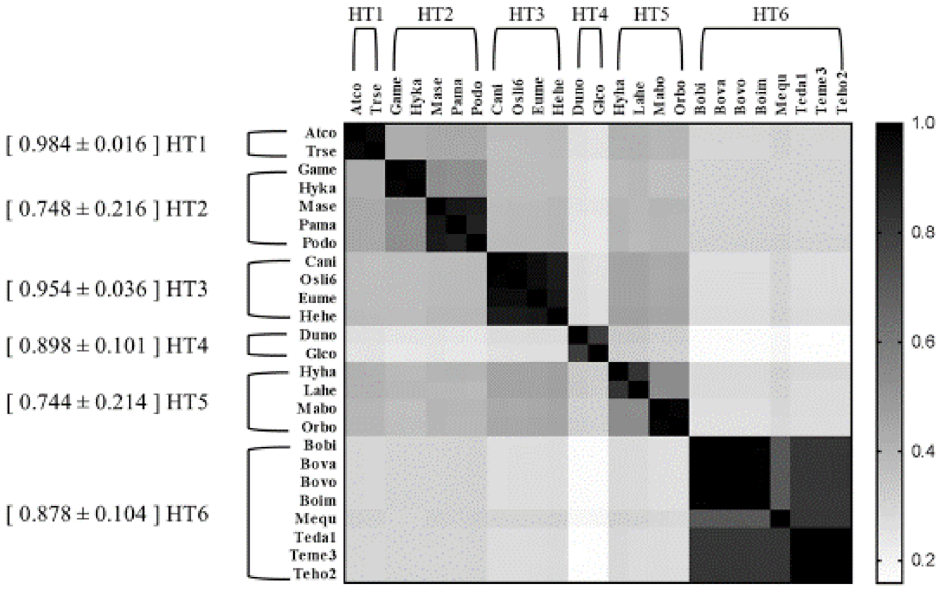

Figure S4: Sequence identities between *Tigger* elements among Arthropoda species. The sequence identities were measured by pairwise comparisons of *Tigger* CDS sequences (for species abbreviations refer to Supplementary table S1).
