# Supplementary material for: Revisiting the Tigger Transposon Evolution Revealing Extensive Involvement in the Shaping of Mammal Genomes
Source: Biology (Basel). 2022 Jun 16;11(6):921. doi: 10.3390/biology11060921 (PMC9219625; doi:10.3390/biology11060921)
Supplement: Supplementary file 1 [file biology-11-00921-s001.zip › biology-1735978-supplementary/supplementary/Supplementary figures S5 & S6.pdf]

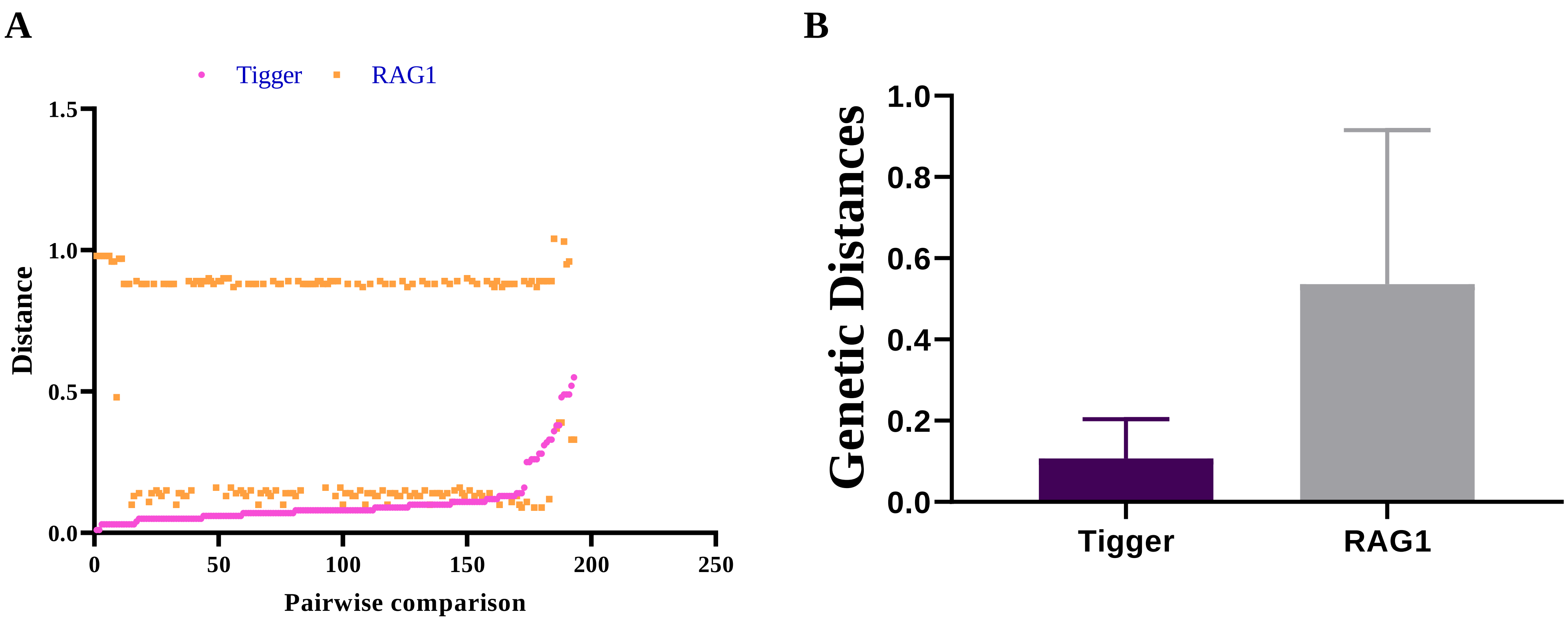

Figure S5: Genetic distances analysis for *Tigger* transposons and host genes. The graph illustrates the pairwise distances of *Tiggers* and RAG1 between vertebrates species that involved in HT events in this study. The distances were obtained from different classes or oders pairwise comparisons between the species in which *Tigger* was identified.

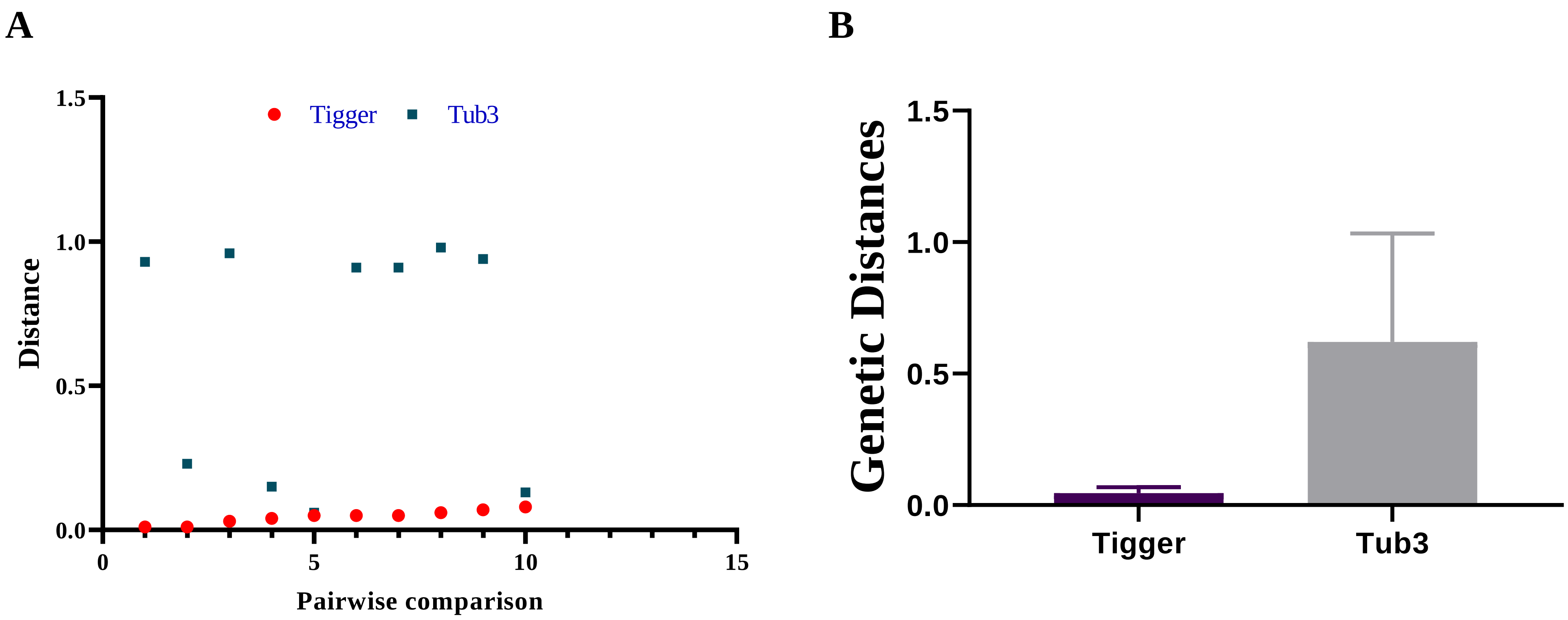

Figure S6: Genetic distances analysis for *Tigger* transposons and host genes. The graph illustrates the pairwise distances of *Tiggers* and tub3 between invertebrates (arthropods) species that involved in HT events in this study. The distances were obtained from different classes or oders pairwise comparisons between the species in which *Tigger* was identified.
