# Supplementary figures and images for: Revisiting the Tigger Transposon Evolution Revealing Extensive Involvement in the Shaping of Mammal Genomes
Source: Biology (Basel). 2022 Jun 16;11(6):921. doi: 10.3390/biology11060921 (PMC9219625; doi:10.3390/biology11060921)

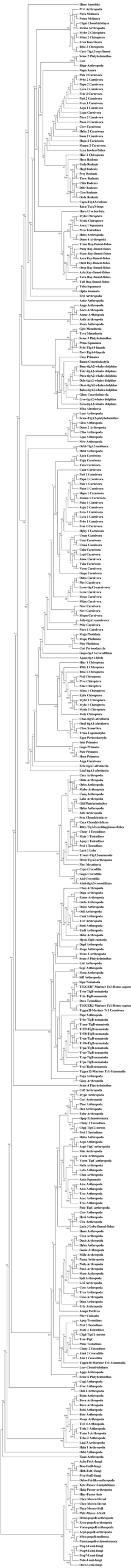

Supplement: Supplementary file 1 [file biology-11-00921-s001.zip › biology-1735978-supplementary/supplementary/Supplementary figure S1.pdf]
